# Comprehensive Evaluation on the Yield, Quality, and Water-Nitrogen Use Efficiency of Mountain Apple Under Surge-Root Irrigation in the Loess Plateau Based on the Improved TOPSIS Method

**DOI:** 10.3389/fpls.2022.853546

**Published:** 2022-04-05

**Authors:** Kun Hao, Liangjun Fei, Lihua Liu, Feilong Jie, Youliang Peng, Xiaogang Liu, Sher Aslam Khan, Dong Wang, Xiukang Wang

**Affiliations:** ^1^State Key Laboratory of Eco-Hydraulics in Northwest Arid Region, Xi’an University of Technology, Xi’an, China; ^2^Institute of Water Resources and Hydroelectric Engineering, Xi’an University of Technology, Xi’an, China; ^3^Faculty of Modern Agricultural Engineering, Kunming University of Science and Technology, Kunming, China; ^4^Department of Plant Breeding and Genetics, The University of Haripur, Haripur, Pakistan; ^5^International Joint Research Laboratory of Global Change Ecology, School of Life Sciences, Henan University, Kaifeng, China; ^6^College of Life Science, Yan’an University, Yan’an, China

**Keywords:** surge-root irrigation, apple, fruit quality, water-nitrogen use efficiency, improved TOPSIS method, fruit yield

## Abstract

The purpose of this study was to know the controlling effects of water and nitrogen coupling on the yield, quality, and water-nitrogen utilization effectiveness of mountain apples under surge-root irrigation in the Loess Plateau. In order to optimize the water and nitrogen irrigation systems of superior quality and high yield, 7 years was selected for the mountain apple test material. The trial was designed with four tiers of irrigation, i.e., full irrigation (FI: 85–100% *θ_*f*_*, where *θ_*f*_* is the field capacity), light deficit irrigation (DI_L_: 70–85% *θ_*f*_*), moderate deficit irrigation (DI_M_: 55–70% *θ_*f*_*), and severe deficit irrigation (DI_S_: 40–55% *θ_*f*_*) and three tiers of nitrogen, i.e., high nitrogen (N_H_: 600 kg ha^–1^), medium nitrogen (N_M_: 400 kg ha^–1^), and low nitrogen (N_L_: 200 kg ha^–1^). The subjective weight attained by the analytic hierarchy methods and the objective weight achieved by the enhanced coefficient of variation method were examined to find the comprehensive weight based on the notion of game hypothesis. Then, the weighted technique for order of preference by similarity to the ideal solution (TOPSIS) process was utilized to comprehensively assess the yield, quality, and water-nitrogen use efficiency of the apples, and a binary quadratic regression model was created between the comprehensive evaluation index and water-nitrogen inputs. The results showed that the effects of irrigation and nitrogen levels on the fruit yield, irrigation water use efficiency (IWUE), total water use efficiency (TWUE), nitrogen partial factor productivity (NPFP), and quality of mountain apples were significant (*P* < 0.05). The apple yield and TWUE first improved and then diminished with an escalating quantity of water-nitrogen inputs, the IWUE diminished with a boost in the irrigation quantity, the NPFP dwindled when the nitrogen amount was increased. The best water and nitrogen inputs for apple yield, quality, or water-nitrogen use efficiency were dissimilar. The best comprehensive evaluation index was DI_L_N_M_ treatment, and the worst comprehensive evaluation index was DI_S_N_L_ treatment, based on the TOPSIS system. The interval of irrigation and nitrogen attained from the mathematic model ranged in 95–115 mm and 470–575 kg ha^–1^, respectively. The outcome of this study may perhaps offer a theoretical basis for the scientific research of surge-root irrigation and the managing of mountain apple tree irrigation and fertilization in the Loess Plateau, China.

## Introduction

Apples (*Malus domestica* Borkh) are one of the most popular fruits in the world, known as the “king of fruits,” and their nutritional and health value is irreplaceable (Kalinowska et al., 2014). China has the largest apple cultivation area and yield in the world, and the Loess Plateau and Bohai Bay are the two largest apple cultivation areas in China. In 2018, the cultivated area and yield of apples in the two major producing areas accounted for 84.22 and 89.12% of the country, respectively, and the Loess Plateau accounted for 65.45 and 53.04% of the country, respectively (Zhou et al., 2021). However, there is a shortage of water resources in the Loess Plateau, and the spatial and temporal distribution of precipitation is uneven, especially in seasonal drought (Suo et al., 2019). At present, local orchards are, for the most part, rain-fed, and developing water-saving irrigation in mountain orchards is urgent. Hence, studying the micro-irrigation technology and water-fertilizer management methods of mountain apple orchards in the Loess Plateau is of the utmost importance.

The two major factors that affect crop growth and development are water and fertilizer. Reasonable regulation of water and fertilizer is one of the foremost ways of improving crop quality and yield (Fentabil et al., 2016; Wang H. D. et al., 2018; Liu et al., 2019). Water-fertilizer coupling technology is an innovative agricultural technology that combines irrigation water and fertilizer solutions and transports it directly to the root zone of crops through the irrigation system (Zhang et al., 2014; Wang Z. H. et al., 2018). Scientific water-fertilizer coupling technology can irrigate and fertilize more precisely, encourage the absorption and consumption of nutrients and water by crops and play a part in regulating fertilizer by water and promoting water by fertilizer.

Coordinating water and fertilizer supply with the demands of crop growth is the solution to improving water and fertilizer utilization efficiency and reducing the cost of production (He et al., 2021). Appropriate water and fertilizer environment is the foundation of the healthy growth, quality, and quantity of crops. Nitrogen is a major element affecting the growth of crops and a major driving force affecting crop yield (Liu et al., 2016; Shah et al., 2017a,2021a; Rose et al., 2018). Extreme nitrogen application can upset the balance between crop reproductive growth and vegetative growth, increase nitrogen loss, and cause environmental pollution (Bai et al., 2019). Too much water and nitrogen application are not conducive to the growth of fruit trees and reduces fruit quality. Sun et al. (2011) considered that too much or too little water and nitrogen supply appreciably lessened the accretion of mineral ferric in apples, while suitable irrigation and nitrogen application can improve the content of trace elements in apple fruits and enhance the nutritional quality of apples. Zhou et al. (2015) found that the content of vitamin C and soluble sugar in apples improved with the increase of nitrogen application rate under moderate deficit irrigation, and the content of titratable acid dropped with the increase of irrigation under the equivalent nitrogen application level; compared with the treatment of the topmost yield, the yield of moderate deficit irrigation under medium nitrogen treatment was not appreciably lessened, but the water use effectiveness was considerably increased. In the past, many studies were done on the outcomes of irrigation or nitrogen applications on the yield of apples and their quality (Raffo et al., 2014; Wang Y. J. et al., 2019; Zhong et al., 2019; Lecaros-Arellano et al., 2021; Wu et al., 2021), but the majority of them focused on just one factor. However, it is unclear whether the correct water nitrogen coupling technology can ensure the quality and yield of mountain apples in the Loess Plateau, and this warrants additional study.

Surge-root irrigation (SRI) is an innovative kind of subsurface infiltration irrigation technology (Fei et al., 2017; He et al., 2020). Proper deficit irrigation under SRI can sponsor fruit growth, improve fruit yield, quality, and water usage efficiency (Qiang et al., 2015; Dai et al., 2019; Zhong et al., 2019). However, hardly any studies have looked into the effect of water-fertilizer coupling technology on fruit trees under SRI, and the effects of water and nitrogen coupling on the yield and quality of mountain apples under SRI in the Loess Plateau is rarely reported. To make up for the dearth of mountain apples in micro-irrigation technology application and water-fertilizer management in the Loess Plateau, in this study, the effects of water and nitrogen input on the yield, quality, and water-nitrogen use efficiency of mountain apples were researched using the water-nitrogen coupling technology under SRI.

Both local and foreign scholars performed wide-ranging assessments on the yield and quality of crops through principal component analysis (Hao et al., 2019; Wang et al., 2020; Li et al., 2021; Xing et al., 2022), analytic hierarchy process (Du et al., 2015; Wang et al., 2021), the technique for order of preference by similarity to the ideal solution (TOPSIS) method (Xiang, 2017; Wang H. D. et al., 2019; Liu et al., 2021), fuzzy comprehensive evaluation (Zhang et al., 2019), gray relational analysis (Wang et al., 2015), and so on. Nevertheless, the evaluation process through a subjective or objective solitary method to determine the weight has its prejudice, as the subjective needs and objective reality cannot be taken into account. Additionally, in the study of crop water and nitrogen utilization, water and nitrogen use efficiency tends to be in a skewed distribution with water and nitrogen input (Liu et al., 2016; Sandhu et al., 2019). When the water and nitrogen use efficiency in the traditional coefficient of variation method is utilized as an assessment index for analysis, its weight must be seriously extreme. Hence, it is hard for domestic and foreign scholars to consistently quantify crop yield, quality, and water-nitrogen use efficiency when exploring them (Wang H. D. et al., 2019). Thus, it is essential to work out the subjective and objective comprehensive weights and ascertain a weighted comprehensive valuation model when optimizing the crop irrigation and fertilization method. Based on this, in the all-inclusive appraisal of this study, the normality test of each evaluation index was completed first, and the index of twisted distribution was changed into an inexact normal distribution. The objective weight was then acquired according to the traditional coefficient of variation method, and the subjective weight was acquired using the analytic hierarchy process. The subjective and objective weights were comprehensively integrated by using the game theory. Lastly, the weighted TOPSIS method was used to expansively appraise the yield, quality, and water and nitrogen utilization efficiency of apples, and the regression model of comprehensive evaluation index and water and nitrogen input was ascertained. The optimal range of irrigation and nitrogen application from the perspective of water saving, fertilizer saving, and quality improvement was then proposed, so as to make available a theoretical basis for the scientific research of SRI and the water and nitrogen management of mountain apples in the Loess Plateau.

## Materials and Methods

### Experimental Site

**FIGURE 1:**
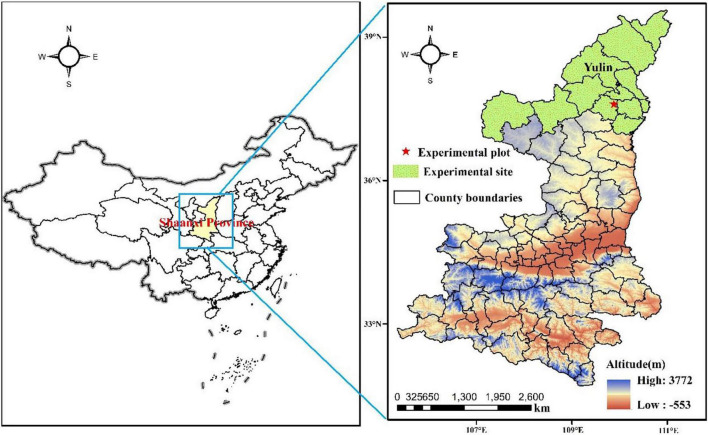
The location of the experimental site.

Field experiments were performed during the apple growing seasons of March–October in 2019 and 2020, at the Zizhou Mountain Apple Demonstration Station of Science and Technology Experiment (37°27′N, 110°2′E, altitude 1,020 m a.s.l.) in Yulin, Shaanxi Province, China. The type of soil in the experimental field is Loessial soil, and its texture was loamy sand (79.26% sand, 20.13% silt, and 0.61% clay) that had a soil bulk density of 1.41 g cm^–3^, a field capacity (volumetric water content) of 21.7%, a pH of 8.3, an organic matter content of 0.81 %, an available N content of 22.6 mg kg^–1^, an available P content of 11.1 mg kg^–1^, and an available K content of 62.3 mg kg^–1^ in the topsoil (0–100 cm). This region is a typical hilly-gully area of the Loess Plateau that has the noteworthy trait of a warm, temperate with a semi-arid climate. The average annual rainfall in the area is only 408.8 mm, the average annual temperature is 9.2°C, the average annual sunshine is 2,632.9 h, with the frost-free period being about 170 days. The rainfall was 415.6 mm and the effective rainfall was 353.0 mm, the daily mean maximum and minimum temperatures were 35.1 and 6.5°C, respectively, from March 20 to October 10, 2019. The rainfall was 423.0 mm and the effective rainfall was 316.5 mm, the daily mean maximum and minimum temperatures were 35.5 and 5.7°C, respectively, from March 20 to October 10, 2020. The location of the experimental site is given in Figure 1.

### Experimental Material

Seven-year-old mountain apple (*M. domestica* Borkh. cv. Hanfu) plants obtained from conventional grafting method were used to graft “Hanfu” bud with Malus robusta Rehd as rootstock and GM256 as interstock with similar growth were chosen as the experimental material. The plant height was 290–315 cm, stem diameter was 9.5–10.6 cm, plant spacing was 2.0 m, and row spacing was 3.0 m (1,667 plants ha^–1^), the planting direction was north-south. Prior to the beginning of the experiment, 10 apple trees were selected randomly in the experimental area, and their root distribution was examined within 200 cm using the root-drilling method (Zhong et al., 2019). The results revealed that more than 90% of the trees’ absorbent roots (< 2 mm in diameter) were dispersed within the depth of 80 cm. The phenological process was split into four main stages (Zhong et al., 2019; Liao et al., 2021): the leaf sprouting stage (I), flowering and fruit-setting stage (II), fruit expansion stage (III), and fruit maturity stage (IV). The precise time divisions are shown in Table 1.

**TABLE 1:** The phenological stage was divided of apple in experimental site.

Year	Sprout leaves stage (I)	Flowering and fruit-setting stage (II)	Fruit expansion stage (III)	Fruit maturity stage (IV)
2019	April 6 to April 29	April 30 to May 20	May 21 to September 16	September 17 to October 8
2020	April 4 to April 24	April 25 to May 18	May 19 to September 21	September 22 to October 10

### Experimental Design and Method

The experiments consisting of irrigation and nitrogen were designed as two factors. A complete combination design was used with 12 treatments (i.e., 4 × 3), and each treatment was applied in 3 plots, with 36 plots in total. The four irrigation levels were full irrigation (FI, 85–100% *θ_*f*_*, where *θ_*f*_* is the field capacity), light deficit irrigation (DI_L_, 70–85% *θ_*f*_*), moderate deficit irrigation (DI_M_, 55–70% *θ_*f*_*), and severe deficit irrigation (DI_S_, 40–55% *θ_*f*_*). Every SRI emitter was used at a system working pressure of 0.1 MPa and a flow rate of 3 L h^–1^, and emitters were installed on both sides of the capillary tube of each apple tree (with the tube located at 40 cm from the tree base and at a buried depth of 40 cm). The irrigation was controlled and measured using a water meter. Irrigation was conducted when the measured soil moisture content of the test plot reached or approached the lower limit designed.

**FIGURE 2:**
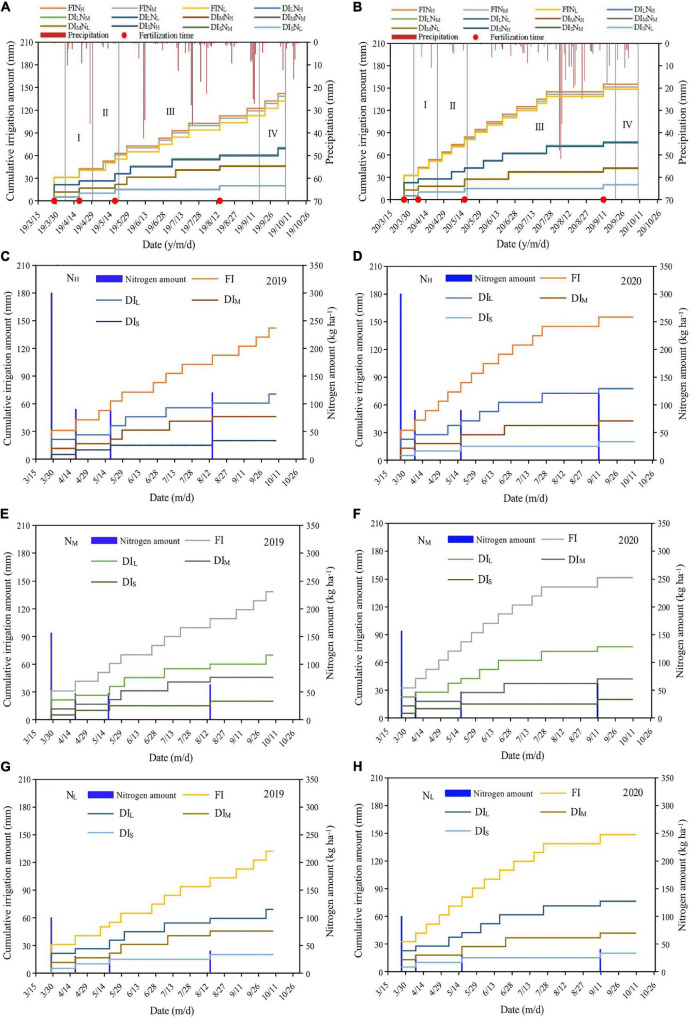
Precipitation, timing, and amounts of irrigation, and nitrogen fertilizer applied each time for apples in 2019 **(A,C,E,G)** and 2020 **(B,D,F,H)**. I, II, III, and IV are the sprout leaves stage, flowering and fruit-setting stage, fruit expansion stage, and fruit maturity stage, respectively. FI, DI_L_, DI_M_, and DI_S_ are full irrigation, light deficit irrigation, moderate deficit irrigation, and severe deficit irrigation, respectively. N_H_, N_M_, and N_L_ are high nitrogen, medium nitrogen, and low nitrogen, respectively.

The irrigation water quota was as follows (Zhong et al., 2019):


(1) $$m=0.1\,\gamma \,z\,p\,S\,\left(\theta_{m\,a\,x}-\theta_{m\,i\,n}\right)/\eta$$

where *m* is the irrigation water quota (L); γ is the soil bulk density (1.41 g cm^–3^); *z* is the planned wetting depth of the soil (0.8 m); *p* is the wetting ratio (0.25; Bai et al., 2001); *S* is the area covered by a single apple tree (6 m^2^); *θ_*max*_* and *θ_*min*_* are the upper and lower limits of the soil mass moisture content, respectively; and η is the coefficient of irrigation water utilization (0.95; Bai et al., 2001). The precipitation and irrigation amounts for each treatment in the experimental periods are illustrated in Figure 2.

In agreement with the local recommended quantity of fertilizer used and prior research experience (Zhang, 2012), three nitrogen levels which were high nitrogen (N_H_, 600 kg ha^–1^), medium nitrogen (N_M_, 400 kg ha^–1^), and low nitrogen (N_L_, 200 kg ha^–1^) were designed. The nitrogen fertilizer was urea (including N 46%). This study applied fixed 240 kg ha^–1^ P_2_O_5_, and 460 kg ha^–1^ K_2_O. The phosphate and potash fertilizers were potassium dihydrogen phosphate (including P_2_O_5_ 52% and K_2_O 34%), and potassium sulfate (including K_2_O 52%), in that order. Before the start of vegetation, 100% of the phosphate fertilizer, 50% of the nitrogen fertilizer, and 33% of the potash fertilizer were applied as the base fertilizer on March 28, 2019 and March 26, 2020, respectively. After that, 15% of the nitrogen fertilizer was applied in stage I (April 18, 2019, and April 7, 2020) and stage II (May 18, 2019, and May 16, 2020), in that order. In addition, 20% of the nitrogen fertilizer and 67% of the potash fertilizer were applied in stage III (August 14, 2019, and September 10, 2020), respectively. The nitrogen, phosphate, and potash fertilizers were dissolved in water then flowed into the soil through an SRI irrigator with a venturi fertilizer applicator. The 2 years of 2019 and 2020 were median water years, and the soil moisture content of DI_S_ failed to make the lesser limit of the experimental design. Consequently, the DI_S_ treatment was irrigated with 5 mm water at each fertilization. Every experimental plot was 10 m long and 3 m wide, and the 3 trees middle were chosen from the 5 trees for the experiment. The entire experimental area was 1,080 m^2^ and consisted of 36 rows spaced 3 m apart. A 1.5 m water separation plate was used to avoid seepage and make certain there was the isolation between experimental plots. The groundwater depth was 25 m; therefore, its effect on the test was insignificant. In addition to water and fertilizer management, cultivation and management measures such as weeding, pest control, and form pruning were identical to those in normal orchard management.

### Plant Sampling and Measurements

#### Soil Moisture Content

**FIGURE 3:**
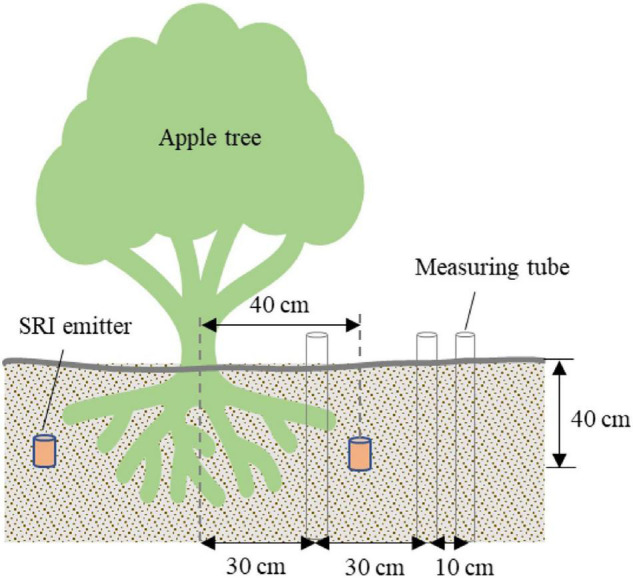
Arrangement of measuring tubes and surge-root irrigation emitters.

A transportable soil moisture meter (TRIME-PICU tubular TDR, IMKO Ltd) was used to quantify the soil’s moisture content. Briefly, 3 measuring tubes were installed at a horizontal distance of 30, 60, and 70 cm from east of the tree base, at horizontal distances of −10, 20, and 30 cm from east of the emitter, and at a depth of 2.0 m (Figure 3). The soil moisture content profile was obtained every 3–5 days. Irrigation was conducted when the measured soil moisture content of the test plot was at or close to the specified lower limit.

#### Yield and Quality

Totally 10–20 days after removing the bags, the apple peel had altered from light yellow to light red, the surface of the apples was smooth, the aroma of the fruit was distinctive, the taste moderately sour and sweet and the flesh was yellow-white, indicating that a number of the apples were ripe. These were harvested in batches during October 5–8, 2019 and October 6–10, 2020, respectively. The yield was measured by weight. A total of 15 apples were selected randomly from each tree, and their quality was determined. The quality indicators were vitamin C, soluble sugar, titratable acid content, sugar-acid ratio, fruit firmness, and color index. Amongst these indicators, the soluble sugar content was determined using 3,5-dinitrosalicylic acid colorimeitry (Negi et al., 2021), the vitamin C content was measured using 2,6-dichloroindophenol sodium indophenol titration (Sun et al., 2022), the titratable acid content was measured using sodium hydroxide (NaOH) titration reference (Liu et al., 2021), the fruit firmness was decided using an FHR-5 fruit firmness tester (Takemura electric works Ltd., toshima-ku, Japan), the color index was established using an SP60 color-spectrophotometer (X-rite Inc., Big Rapids, MI, United States). The sugar–acid ratio is the ratio of soluble sugar content to titratable acid content (Liu et al., 2021).

#### Water Consumption

Water consumption was estimated using the water balance method:


(2) $$E\,T_{i}=I_{i}+P_{r}-R_{f}-D+U+W_{0}-W_{f}$$

where *ET*_*i*_, *I*_*i*_, *P*_*r*_, *R*_*f*_, *D*, and *U* are the water consumption of the fruit trees in the period (mm), irrigation water in the period (mm), effective rainfall (mm), surface runoff (mm), deep leakage (mm), and groundwater recharge in the period (mm), respectively; *W*_0_ and *W*_*f*_ indicate the water stored in the soil at the start and end of the period, respectively (mm).

The effective rainfall is simplified to the product of rainfall and the coefficient of effective precipitation utilization in production practice (Wang, 2009).


(3) $$P_{r}=\sigma \,P$$

where *P* and σ are the rainfall (collected by small weather stations; mm) and coefficient of effective precipitation utilization, respectively. When *P* < 5 mm, σ = 0; when 5 ≤ *P* < 50 mm, σ = 1.0; and when *P* ≥ 50 mm, σ = 0.75.

Due to the small outflow and low irrigation quota, the surface runoff and deep leakage caused by irrigation could be ignored (*R_*f*_* = 0 and *D* = 0). In addition, the groundwater level in the test area was less than 25 m, so the groundwater recharge was not taken into consideration (*U* = 0).


(4) $$E\,T_{i}=I_{i}+\sigma \,P+W_{0}-W_{f}$$

#### Water-Nitrogen Use Efficiency

The irrigation water use efficiency (IWUE, kg m^–3^) is the ratio of the yield to total irrigation amount:


(5) IWUE=0.1⁢Y/I

where *Y* is the yield (kg ha^–1^), and *I* is the total irrigation amount (mm).

The total water use efficiency (TWUE, kg m^–3^) is the ratio of the yield to total water consumption (*ET*_*i*_, mm):


(6) $$\text{TWUE}=0.1\,Y/\Sigma \,E\,T_{i}$$

The nitrogen partial factor productivity (NPFP, kg kg^–1^) was calculated as:


(7) NPFP=Y/N

where *N* is the nitrogen amount (kg ha^–1^).

### Basic Principles and Procedure of Improved TOPSIS Method

(1) Build the comprehensive evaluation indicator system.
(2) The data matrix *R* of the evaluation objects and evaluation indicators was established: there were 4 × 3 (four irrigation levels and three nitrogen levels) evaluation objects and ten (fruit yield, IWUE, TWUE, NPFP, soluble sugar, vitamin C, titratable acid, sugar-acid ratio, fruit firmness, and color index) evaluation indicators:

(8) $$\text{R}={\left(r_{i\,j}\right)}_{m\times n}$$

where *r*_*ij*_ is the original data of the *j*th evaluation index in the *i*th evaluation sample, with *m* = 12 and *n* = 10.
(3) The combination weighting method of game theory was used to determine the index weight *W*:
(a) The subjective weight *W*_1_ was obtained by the analytic hierarchy process, the specific steps refer to the researchers of Nilsson et al. (2016) and Wang et al. (2021).
(b) The objective weight *W*_2_ was obtained by improving the coefficient of variation method.
(i) The normality test of each evaluation index was carried out, and the index of twisted distribution was transformed into an estimated normal distribution. In this paper, the IWUE and NPFP index becomes a grave bias distribution because of the noticeable disparity between irrigation amount and nitrogen amount gradient. If the original data were analyzed directly, their weight would become a great deal larger. We transform the IWUE and NPFP into rough normal distribution by logarithmic transformation method to form a new decision matrix *R’*.

(9) $${\text{R}}^{\prime }={\left(r_{i\,j}^{\prime }\right)}_{m\times n}$$

(ii) *R’* was standardized to obtain the normalized decision-making matrix *Z* = (*z*_*ij*_)*_*m*_*_×_ *_*n*_* :

(10) $$z_{i\,j}=r_{i\,j}^{\prime }\cdot {\left(\sum_{i=1}^{n}{\left(r_{i\,j}^{\prime }\right)}^{2}\right)}^{-0.5}$$

(iii) The coefficient of variation ***V*** was calculated:

(11) $$v_{j}=\frac{\sigma_{j}}{{\bar{x}}_{j}}$$

where *v*_*j*_, σ_*j*_, ${\bar{x}}_{j}$ are coefficient of variation, standard deviation, and the average value of the *j*th evaluation index in the normalized decision-making matrix, respectively.
(iv) The coefficient of variation *V* was normalized to obtain *W*_2_.

(12) $$w_{j}=v_{j}\cdot {\left(\sum_{j=1}^{n}v_{j}\right)}^{-1}$$

(c) The principle of game theory was used for combination weighting.
(i) Construct basic weight vector ***u**_*k*_* = {***u**_*k*_*_1_, ***u**_*k*_*_2_} (*k* = 1, 2), then the ***u**_*k*_* was linearly combined:

(13) $$\text{u}=\alpha_{1}\,{\text{u}}_{1}+\alpha_{2}\,{\text{u}}_{2}$$

where *u* is a possible weight vector of the *u*_*k*_, α_1_ and α_2_ are linear combination coefficient, respectively, α_1_ > 0, α_2_ > 0, α_1_***+***α_2_ = 1.
(ii) In order to minimize the deviation between ***u*** and ***u*****_1,_** and ***u*****_2_**, the idea of game theory was used to optimize the linear combination coefficient α_1_ and α_2_.

(14) $$\begin{array}{cc} \min={||\left(\alpha_{1}\,u_{1}^{T}+\alpha_{2}\,u_{2}^{T}\right)-u_{k}||}_{2} & \left(k=1,2\right) \end{array}$$

(iii) The optimal first derivative condition of Equation 13 can be transformed into a system of equations:

(15) $$\left[\begin{array}{cc} u_{1}\cdot u_{1}^{T} & u_{1}\cdot u_{2}^{T}\\ u_{2}\cdot u_{1}^{T} & u_{2}\cdot u_{2}^{T} \end{array}\right]\,\left[\begin{array}{c} \alpha_{1}\\ \alpha_{2} \end{array}\right]=\left[\begin{array}{c} u_{1}\cdot u_{1}^{T}\\ u_{2}\cdot u_{2}^{T} \end{array}\right]$$

(iv) The combined weight vector was calculated after α_1_ and α_2_ were calculated from Equation 14.

(16) $$W=\alpha_{1}\,W_{1}+\alpha_{2}\,W_{2}$$

(4) The weight normalized data matrix *Z”* = *WZ* was established.
(5) The positive ideal solution *Z*^+^ and the negative ideal solution *Z*^–^ were calculated.

(17) $$z_{j}^{+}=\left\{ \begin{array}{c} \begin{array}{cc} \max_{1\leq i\leq n}z_{i\,j}^{\prime \prime } & B\,e\,n\,e\,f\,i\,t\,t\,y\,p\,e\,a\,t\,t\,r\,i\,b\,u\,t\,e \end{array}\\ \begin{array}{cc} \min_{1\leq i\leq n}z_{i\,j}^{\prime \prime } & C\,o\,s\,t\,t\,y\,p\,e\,a\,t\,t\,r\,i\,b\,u\,t\,e \end{array} \end{array}\right.$$

(18) $$z_{j}^{-}=\left\{ \begin{array}{c} \begin{array}{cc} \min_{1\leq i\leq n}z_{i\,j}^{\prime \prime } & B\,e\,n\,e\,f\,i\,t\,t\,y\,p\,e\,a\,t\,t\,r\,i\,b\,u\,t\,e \end{array}\\ \begin{array}{cc} \max_{1\leq i\leq n}z_{i\,j}^{\prime \prime } & C\,o\,s\,t\,t\,y\,p\,e\,a\,t\,t\,r\,i\,b\,u\,t\,e \end{array} \end{array}\right.$$

(6) Calculate the Euclidean distances *D*^+^ and *D*^–^ between each evaluation object and *Z*^+^ and *Z*^–^.

(19) $$d_{i}^{+}={\left(\sum_{i=1}^{m}{\left(z_{i\,j}^{\prime \prime }-z_{j}^{+}\right)}^{2}\right)}^{0.5}$$

(20) $$d_{i}^{-}={\left(\sum_{i=1}^{m}{\left(z_{i\,j}^{\prime \prime }-z_{j}^{-}\right)}^{2}\right)}^{0.5}$$

(7) Calculate the comprehensive evaluation index, that is, the proximity ***F*** between each evaluation object and the optimal scheme, and then sort it. If *f*_*i*_ was closer to 1, it means that the evaluation object was better.

(21) $$f_{i}=\frac{d_{i}^{-}}{d_{i}^{+}+d_{i}^{-}},\left(0\leq f_{i}\leq 1\right)$$

### Statistical Analysis

The data statistical analysis was carried out using Excel (Version 2013, Microsoft Corp., Redmond, WA, United States), and data plotting was executed using Matlab (Version 9.4, MathWorks Inc., Natick, MA, United States) and Origin (Version 9.0, OriginLab Corp., Northampton, MA, United States) software, while correlation analysis and variance analysis were completed using the IBM SPSS software (Version 21.0, Armonk, NY, United States). If the measurement variables conform to the normal distribution and variance homogeneity, the analysis of variance was used, and the treatment means were compared for any major differences using Duncan’s multiple range tests at the *P* = 0.05 level.

## Results

### Fruit Yield

**FIGURE 4:**
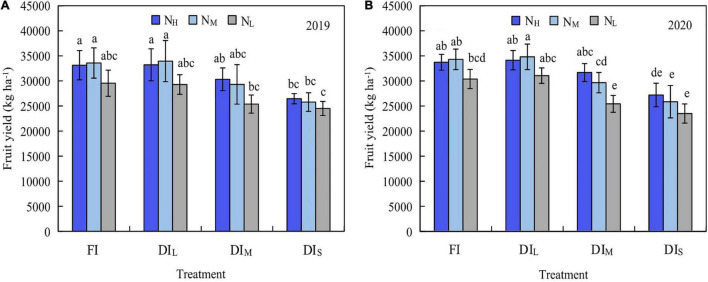
Effects of water and nitrogen coupling on yield of mountain apple in 2019 **(A)** and 2020 **(B)**. Different letters above the bars indicate a significant difference at *P* < 0.05 according to the Duncan test. FI, DI_L_, DI_M_, and DI_S_ are full irrigation, light deficit irrigation, moderate deficit irrigation, and severe deficit irrigation, respectively. N_H_, N_M_, and N_L_ are high nitrogen, medium nitrogen, and low nitrogen, respectively. Bars and errors stand to represent mean ± SD.

The effects of irrigation level and nitrogen level on apple yield were significant (*P* < 0.05; Figure 4). The yield initially increased and then decreased with an increasing nitrogen quantity under FI and DI_L_, while under DI_M_ and DI_S_, it increased with a boost in the nitrogen amount. Under the identical nitrogen level, the yield initially increased and then decreased with an increasing irrigation amount. The largest apple yield was achieved under DI_L_N_M_ treatment, reaching 33,955 kg ha^–1^ in 2019 and 34,817 kg ha^–1^ in 2020. The DI_S_N_L_ treatment had the least yield, with only 24,509 kg ha^–1^ in 2019 and 23,508 kg ha^–1^ in 2020. In comparison with the DI_S_N_L_ treatment, the other treatments improved the yield by 3.54–38.48% and 8.23–48.16% in 2019 and 2020, respectively.

### Water-Nitrogen Use Efficiency

**FIGURE 5:**
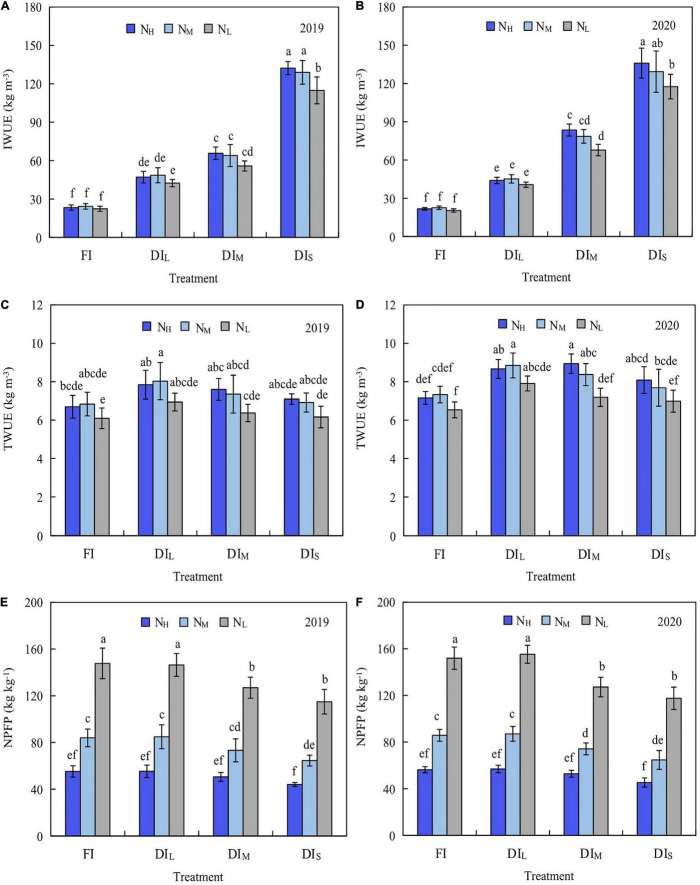
Effects of water and nitrogen coupling on irrigation water use efficiency (2019, **A**; 2020, **B**), total water use efficiency (2019, **C**; 2020, **D**), and nitrogen partial factor productivity (2019, **E**; 2020, **F**) of mountain apple. Different letters above the bars indicate a significant difference at *P* < 0.05 according to the Duncan test. FI, DI_L_, DI_M_, and DI_S_ are full irrigation, light deficit irrigation, moderate deficit irrigation, and severe deficit irrigation, respectively. N_H_, N_M_, and N_L_ are high nitrogen, medium nitrogen, and low nitrogen, respectively. Bars and errors stand to represent mean ± standard deviation.

The effects of irrigation level and nitrogen level on IWUE, TWUE, and NPFP were significant (*P* < 0.05; Figure 5). Under the FI and DI_L_, there was no significant difference in IWUE and TWUE among treatments (*P* > 0.05), while the IWUE and TWUE increased with an increase in the amount of nitrogen under DI_M_ and DI_S_. Under the same nitrogen level, the IWUE and TWUE diminished with a rise in the irrigation quantity. In comparison with DI_S_N_L_ treatment, the DI_S_N_H_ and DI_S_N_M_ treatments increased the IWUE by 15.18 and 12.28%, respectively, but the other treatments reduced the IWUE by 42.77–80.52% in 2019, the DI_S_N_H_ and DI_S_N_M_ treatments increased the IWUE by 15.68% and 10.01%, in that order, but the additional treatments reduced the IWUE by 28.91–82.60% in 2020; the FIN_L_ treatment reduced the TWUE by 1.14%, while the other treatments boosted the TWUE by 3.41–30.36% in 2019, among them, the DI_L_N_M_ treatment increased the TWUE by 30.36%; the FIN_L_ treatment decreased the TWUE by 6.58%, but the other treatments increased the TWUE by 2.29–27.90% in 2020, among them, the DI_M_N_M_ treatment achieved the largest TWUE, followed by DI_L_N_M_ treatment, and the TWUE of DI_M_N_M_ and DI_L_N_M_ treatments had no significant difference (*P* > 0.05).

Under the equivalent irrigation level, the NPFP diminished with an increase in the amount of nitrogen. There was no major disparity in NPFP amongst the treatments (*P* > 0.05) under the N_H_, whereas the NPFP increased trend with an increase in the irrigation quantity under N_M_ and N_L_, and the FI and DI_L_ had no significant divergence (*P* > 0.05). In comparison with the DI_S_N_L_ treatment, the FIN_L_, DI_L_N_*L*,_ and DI_M_N_L_ treatments increased the NPFP by 28.63, 27.49, and 10.53%, respectively, but the other treatments decreased the NPFP by 26.09–61.61% in 2019; the FIN_L_, DI_L_N_*L*,_ and DI_M_N_L_ treatments increased the NPFP by 29.28, 32.13, and 8.21%, respectively, but the other treatments diminished the NPFP by 25.95–61.44% in 2020.

### Fruit Quality

The flavor and product value of apples are broadly affected by numerous quality pointers, and the dissimilarities between these indicators are revealed in Table 2. The effects of irrigation levels and nitrogen levels on apple quality were significant (*P* < 0.05). The soluble sugar content, sugar-acid ratio, and color index of the DI_L_N_M_ treatment were the biggest, reaching 10.67%, 27.67, and 4.31 in 2019 and 10.64 %, 27.00, and 4.36 in 2020; whereas the DI_S_N_L_ treatment was the least. The vitamin C content of the FIN_H_ treatment was the biggest, reaching 4.52 and 4.46 mg (100 g)^–1^ in 2019 and 2020, respectively, whereas the DI_S_N_L_ treatment had the lowest vitamin C content. The titratable acid content of DI_L_N_M_ treatment was the lowest, at only 0.39 and 0.40% in 2019 and 2020, respectively, while the DI_S_N_L_ treatment was the biggest, at 0.50 and 0.50 % in 2019 and 2020, respectively. The fruit firmness of DI_S_N_L_ treatment was the largest, at 8.44 and 8.51 kg cm^–2^ in 2019 and 2020, in that order, where the FIN_H_ treatment was the least, at only 7.62 and 7.73 kg cm^–2^ in 2019 and 2020, respectively.

**TABLE 2:** Effects of water and nitrogen coupling on quality of mountain apple.

Year	Irrigation level	Nitrogen level	Soluble sugar/%	Vitamin C /[mg (100 g)^–1^]	Titratable acid/%	Sugar acid ratio	Fruit firmness /(kg cm^–2^)	Color index
2019	FI	N_H_	10.27 ± 0.18bcd	4.52 ± 0.06a	0.413 ± 0.040de	25.03 ± 2.91abc	7.62 ± 0.08e	4.22 ± 0.13ab
		N_M_	10.53 ± 0.28ab	4.36 ± 0.04ab	0.397 ± 0.025de	26.66 ± 2.39ab	7.85 ± 0.12cde	4.29 ± 0.11ab
		N_L_	9.76 ± 0.22fg	4.18 ± 0.09cde	0.440 ± 0.036abcde	22.27 ± 1.85cde	7.97 ± 0.21cd	4.10 ± 0.14abc
	DI_L_	N_H_	10.39 ± 0.16abc	4.47 ± 0.12a	0.407 ± 0.031de	25.66 ± 2.27abc	7.71 ± 0.10de	4.25 ± 0.20ab
		N_M_	10.67 ± 0.20a	4.25 ± 0.13bcd	0.387 ± 0.021e	27.67 ± 1.91a	7.89 ± 0.09cde	4.31 ± 0.15a
		N_L_	9.72 ± 0.17fg	4.09 ± 0.05de	0.433 ± 0.031bcde	22.52 ± 2.00cde	8.07 ± 0.26bc	4.08 ± 0.17abcd
	DI_M_	N_H_	10.03 ± 0.16def	4.28 ± 0.07bc	0.440 ± 0.026abcde	22.86 ± 1.68bcde	8.02 ± 0.17bc	4.01 ± 0.21bcd
		N_M_	10.16 ± 0.27cde	4.13 ± 0.11cde	0.420 ± 0.030cde	24.30 ± 2.37abcd	8.27 ± 0.09ab	3.94 ± 0.11cd
		N_L_	9.68 ± 0.19fg	3.89 ± 0.08f	0.473 ± 0.021abc	20.50 ± 1.31de	8.39 ± 0.16a	3.57 ± 0.15ef
	DI_S_	N_H_	9.83 ± 0.10efg	4.05 ± 0.14e	0.447 ± 0.035abcd	22.10 ± 1.99cde	8.26 ± 0.14ab	3.81 ± 0.12de
		N_M_	9.74 ± 0.12fg	3.84 ± 0.09fg	0.480 ± 0.040ab	20.40 ± 1.96de	8.37 ± 0.11a	3.66 ± 0.07e
		N_L_	9.52 ± 0.18g	3.69 ± 0.07g	0.497 ± 0.025a	19.21 ± 1.35e	8.44 ± 0.10a	3.37 ± 0.16f
2020	FI	N_H_	10.19 ± 0.12bcd	4.46 ± 0.09a	0.427 ± 0.031def	23.97 ± 1.91bc	7.73 ± 0.16g	4.23 ± 0.14abc
		N_M_	10.44 ± 0.21ab	4.29 ± 0.12bc	0.413 ± 0.015ef	25.29 ± 1.39ab	7.91 ± 0.13efg	4.31 ± 0.12ab
		N_L_	9.82 ± 0.17efg	4.15 ± 0.04cdef	0.450 ± 0.020bcde	21.87 ± 1.33cde	8.04 ± 0.09def	4.09 ± 0.21abcd
	DI_L_	N_H_	10.31 ± 0.25abc	4.39 ± 0.06ab	0.420 ± 0.026def	24.62 ± 2.03abc	7.78 ± 0.14fg	4.27 ± 0.09abc
		N_M_	10.64 ± 0.21a	4.22 ± 0.11cde	0.397 ± 0.035f	27.00 ± 2.93a	7.95 ± 0.24efg	4.36 ± 0.16a
		N_L_	9.87 ± 0.20defg	4.01 ± 0.07fgh	0.457 ± 0.025abcde	21.66 ± 1.60cde	8.16 ± 0.12bcde	4.01 ± 0.23cde
	DI_M_	N_H_	9.98 ± 0.20cdef	4.26 ± 0.08bcd	0.463 ± 0.023abcd	21.59 ± 1.47cde	8.14 ± 0.19cde	4.04 ± 0.14bcd
		N_M_	10.09 ± 0.17cde	4.07 ± 0.10efgh	0.440 ± 0.026cdef	23.00 ± 1.79bcd	8.36 ± 0.20abc	3.89 ± 0.11de
		N_L_	9.74 ± 0.21efg	3.93 ± 0.10h	0.487 ± 0.012ab	20.03 ± 0.88de	8.45 ± 0.16abc	3.51 ± 0.15fg
	DI_S_	N_H_	9.81 ± 0.17efg	4.11 ± 0.06defg	0.473 ± 0.015abc	20.75 ± 1.03de	8.31 ± 0.13abcd	3.74 ± 0.16ef
		N_M_	9.69 ± 0.10fg	3.97 ± 0.07gh	0.487 ± 0.021ab	19.93 ± 0.69de	8.46 ± 0.15ab	3.58 ± 0.17fg
		N_L_	9.58 ± 0.21g	3.72 ± 0.12i	0.500 ± 0.030a	19.23 ± 1.58e	8.51 ± 0.21a	3.32 ± 0.12g

*FI, DI_L_, DI_M_, and DI_S_ are full irrigation, light deficit irrigation, moderate deficit irrigation, and severe deficit irrigation, respectively. N_H_, N_M_, and N_L_ are high nitrogen, medium nitrogen, and low nitrogen, respectively. Data is mean ± standard deviation (n = 3). Different small letters in the same column indicated significant difference at 0.05 level according to the Duncan test in the same year.*

### Correlation Analysis

The results of the Pearson correlation analysis of the yield, water-nitrogen use efficiency, and quality of apples (Table 3) showed significant positive correlations between fruit yield and soluble sugar, vitamin C, and sugar-acid ratio, as well as the color index. Fruit yield was negatively associated with IWUE, titratable acid content, and fruit firmness. Significant positive correlations were discerned between IWUE and titratable acid content, and fruit firmness. IWUE was negatively associated with vitamin C content, sugar-acid ratio, and color index. Significant positive correlations between soluble sugar content and vitamin C content, sugar-acid ratio, and color index. Soluble sugar content was negatively correlated with titratable acid content, and firmness of the fruit. Significant positive correlations were found between vitamin C content and color index. Vitamin C content was negatively correlated with a titratable acid content, and fruit firmness. Significant positive correlations between titratable acid content and fruit firmness; Titratable acid content was negatively correlated with sugar-acid ratio and color index. There were significant positive correlations between sugar-acid ratio and color index, while the fruit firmness negatively correlated with either sugar-acid ratio or color index. Additionally, significant positive correlations were found between TWUE and soluble sugar content, and sugar-acid ratio in 2019, with significant negative correlations between IWUE and soluble sugar content in 2020. Thus, it can be concluded that the comprehensive appraisal of the yield, water-nitrogen use efficiency, and quality of the apples cannot be scientifically conducted merely through analysis of the relationships between indicators.

**TABLE 3:** Correlation analysis of yield, water-nitrogen use efficiency, and quality of apple.

	Fruit yield	IWUE	TWUE	NPFP	Soluble sugar	Vitamin C	Titratable acid	Sugar acid ratio	Fruit firmness	Color index
Fruit yield	1	−0.762**	0.394	−0.227	0.894**	0.882**	−0.941**	0.914**	−0.923**	0.991**
IWUE	−0.736**	1	0.164	−0.337	−0.601*	−0.606*	0.722**	−0.669*	0.784**	−0.771**
TWUE	0.555	−0.026	1	−0.547	0.471	0.315	−0.354	0.394	−0.153	0.342
NPFP	−0.271	−0.316	−0.597*	1	−0.326	−0.501	0.215	−0.245	0.214	−0.188
Soluble sugar	0.908**	−0.507	0.685*	−0.464	1	0.739**	−0.964**	0.988**	−0.768**	0.872**
Vitamin C	0.924**	−0.707*	0.480	−0.392	0.786**	1	−0.788**	0.746**	−0.914**	0.880**
Titratable acid	−0.948**	0.673*	−0.617*	0.265	−0.915**	−0.870**	1	−0.990**	0.847**	−0.940**
Sugar acid ratio	0.949**	−0.613*	0.648*	−0.336	0.973**	0.835**	−0.981**	1	−0.810**	0.904**
Fruit firmness	−0.928**	0.735**	−0.369	0.246	−0.739**	−0.950**	0.817**	−0.796**	1	−0.922**
Color index	0.959**	−0.736**	0.525	−0.189	0.820**	0.916**	−0.951**	0.908**	−0.906**	1

IWUE, TWUE, and NPFP are irrigation water use efficiency, total water use efficiency, and nitrogen partial factor productivity, respectively. ** and * represent P < 0.01 and P < 0.05, respectively. The lower left part and upper right part are the correlation analysis of 2019 and 2020, respectively.

### Comprehensive Evaluation by Improved TOPSIS Method

It can be found from Figures 4, 5 and Table 3, that the most favorable inputs of water and nitrogen for apple yield, quality, or water-nitrogen use efficiency are dissimilar. Thus, it is imperative to establish a comprehensive appraisal system for apple yield, quality, and water-nitrogen use efficiency. In this research, the game theory combined weights TOPSIS method was utilized to expansively assess apple yield, quality, and water-nitrogen use efficiency. As shown in Table 4, the analytic hierarchy process demonstrated that the subjective weights of apple yield, WUE, NPFP, and quality are 0.457, 0.146, 0.094, and 0.303, in that order. The improved coefficient of variation method found that in 2019, the objective weights of apple yield, WUE, NPFP, and quality are 0.134, 0.289, 0.112, and 0.465, respectively, while in 2020, they were 0.146, 0.311, 0.110, and 0.433, respectively. The game theory combined weights process demonstrated that the combined weights of apple yield, WUE, NPFP, and quality are 0.429, 0.158, 0.096, and 0.317, in that order, in 2019 and in 2020 are 0.429, 0.161, 0.095, and 0.315, respectively. As shown in Table 5, the ranking of comprehensive evaluation indexes based on the TOPSIS method in 2019 and 2020 is fundamentally identical from high to low, the top 4 are DI_L_N_M_, FIN_M_, DI_L_N_*H*,_ and FIN_H_ treatments, respectively, and the last was DI_S_N_L_ treatment. Significant correlations were found between the comprehensive evaluation index and most evaluation index (Table 4), which demonstrates that determining the amount of water and nitrogen input using the game theory combined weights TOPSIS method is reliable.

**TABLE 4:** Weight and ideal solution of the TOPSIS method, and correlation coefficient between the comprehensive evaluation index and each evaluation index.

		Fruit yield	IWUE	TWUE	NPFP	Soluble sugar	Vitamin C	Titratable acid	Sugar acid ratio	Fruit firmness	Color index
Subjective weight		0.4564	0.0365	0.1096	0.0943	0.1035	0.0784	0.0235	0.0482	0.0312	0.0184
Objective weight	2019	0.1344	0.1845	0.1040	0.1123	0.0433	0.0706	0.0905	0.1313	0.0395	0.0896
	2020	0.1457	0.1962	0.1148	0.1098	0.0369	0.0573	0.0819	0.1214	0.0377	0.0983
Combination weight	2019	0.4294	0.0489	0.1091	0.0958	0.0985	0.0778	0.0291	0.0552	0.0319	0.0243
	2020	0.4287	0.0508	0.1100	0.0957	0.0976	0.0765	0.0287	0.0547	0.0318	0.0255
Positive ideal solution	2019	0.1416	0.0171	0.0360	0.0314	0.0302	0.0244	0.0074	0.0188	0.0096	0.0076
	2020	0.1418	0.0177	0.0362	0.0315	0.0299	0.0238	0.0073	0.0189	0.0096	0.0081
Negative ideal solution	2019	0.1022	0.0109	0.0273	0.0238	0.0270	0.0200	0.0095	0.0131	0.0087	0.0059
	2020	0.0957	0.0109	0.0264	0.0238	0.0269	0.0199	0.0092	0.0135	0.0087	0.0062
*R*	2019	0.996**	−0.724**	0.586*	−0.257	0.908**	0.904**	−0.943**	0.947**	−0.911**	0.946**
	2020	0.993**	−0.743**	0.444	−0.211	0.889**	0.849**	−0.932**	0.908**	−0.902**	0.981**

IWUE, TWUE, and NPFP are irrigation water use efficiency, total water use efficiency, and nitrogen partial factor productivity, respectively. R is the Pearson correlation coefficient between the comprehensive evaluation index and each evaluation index. *P < 0.05 and **P < 0.01.

**TABLE 5:** Comprehensive evaluation index on yield, quality, and water-nitrogen use efficiency of mountain apple under water and nitrogen coupling by TOPSIS method.

Irrigation level	Nitrogen level	2019				2020			
		Positive ideal distance	Negative ideal distance	Comprehensive evaluation index	Ranking	Positive ideal distance	Negative ideal distance	Comprehensive evaluation index	Ranking
FI	N_H_	0.0113	0.0367	0.7639	4	0.0128	0.0422	0.7676	4
	N_M_	0.0090	0.0389	0.8118	3	0.0103	0.0448	0.8136	3
	N_L_	0.0218	0.0226	0.5093	8	0.0222	0.0292	0.5690	7
DI_L_	N_H_	0.0080	0.0379	0.8250	2	0.0083	0.0448	0.8434	2
	N_M_	0.0052	0.0413	0.8883	1	0.0056	0.0479	0.8958	1
	N_L_	0.0211	0.0220	0.5108	7	0.0172	0.0325	0.6537	6
DI_M_	N_H_	0.0173	0.0259	0.5993	5	0.0152	0.0353	0.6986	5
	N_M_	0.0205	0.0219	0.5166	6	0.0221	0.0270	0.5501	8
	N_L_	0.0373	0.0085	0.1857	11	0.0395	0.0115	0.2252	11
DI_S_	N_H_	0.0328	0.0116	0.2618	9	0.0326	0.0179	0.3542	9
	N_M_	0.0354	0.0094	0.2099	10	0.0378	0.0129	0.2550	10
	N_L_	0.0411	0.0084	0.1690	12	0.0475	0.0089	0.1581	12

*FI, DI_L_, DI_M_, and DI_S_ are full irrigation, light deficit irrigation, moderate deficit irrigation, and severe deficit irrigation, respectively. N_H_, N_M_, and N_L_ are high nitrogen, medium nitrogen, and low nitrogen, respectively.*

### Relationship of Comprehensive Evaluation Index With the Amounts of Water and Nitrogen

A binary quadratic regression equation was established by irrigation amount and nitrogen application rate being treated as the independent variables, and the comprehensive evaluation index is considered as the response variables.


$$F_{1}=-0.779+1.69\times 10^{-2}\,I_{1}+3.13\times 10^{-3}\,N+2.69$$

$$\times 10^{-6}\,I_{1}\,N-8.62\times 10^{-5}\,I_{1}^{2}-3.27\times 10^{-6}\,N^{2}$$

(22) $$\left(R^{2}=0.92,F=13.25,P<0.01\right)$$

F2=-0.678+1.78×10⁢I2-2+2.65×10-3⁢N-1.33

$$\times 10^{-6}\,I_{2}\,N-8.06\times 10^{-5}\,I_{2}^{2}-2.34\times 10^{-6}\,N^{2}$$

(23) $$\left(R^{2}=0.95,F=24.30,P<0.01\right)$$

where *F*_1_ and *F*_2_ are the comprehensive evaluation index in 2019 and 2020, respectively. *I*_1_ and *I*_2_ are the total irrigation amount in 2019 and 2020, respectively (2019: 20–142.10 mm; 2020: 20–155.11 mm). *N* is the nitrogen amount (200–600 kg ha^–1^).

**FIGURE 6:**
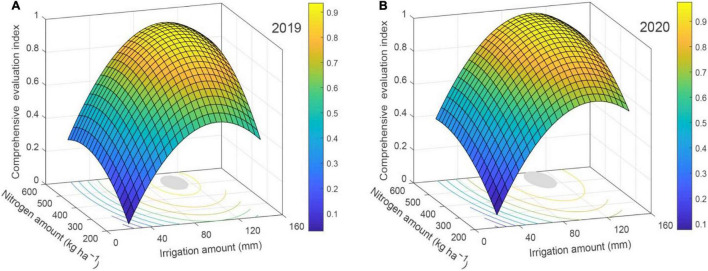
Coupling effects of water and nitrogen on comprehensive evaluation index of mountain apple in 2019 **(A)** and 2020 **(B)**.

The comprehensive evaluation index of apples confirmed an opening downward paraboloid with the input of irrigation amount and nitrogen amount (Figure 6). The comprehensive evaluation index first increased and then reduced with escalating nitrogen amount when the quantity of irrigation was unvarying. The comprehensive evaluation index also initially grew and then diminished with increasing irrigation quantity when the nitrogen application rate was constant. It can be seen from Equations 22, 23, and Figure 6, that the optimum comprehensive evaluation index of 0.94 was realized when 106.18 mm of irrigation quantity and fertilization rate of 522.27 kg N ha^–1^ were applied in 2019, and the comprehensive evaluation index was maximized at 0.98 with the irrigation quantity of 106.00 mm and 536.12 kg N ha^–1^ in 2020. When the comprehensive evaluation index reached 99% of the maximum value, the irrigation amount interval and the nitrogen amount interval were 95.76–116.59 mm and 466.78–575.75 kg ha^–1^ in 2019, and 95.00–117.00 mm and 471.56–600.68 kg ha^–1^ in 2020, in that order.

## Discussion

### Coupling Effects of Water and Nitrogen on Apple Yield and Water-Nitrogen Use Efficiency

Unreasonable regulation of water and fertilizer will rigorously limit the growth of crops, resulting in lesser yields, poor quality, and even death (Li et al., 2020; Shah et al., 2021b). An appropriate soil water and nitrogen situation will improve the nutrient absorption ability of crop roots, thus increasing yield and water and nitrogen utilization (Liu et al., 2016; Padilla et al., 2017). When both the vegetative growth and reproductive growth of fruit trees develop in an impartial manner, increased water and nitrogen application can attain higher water and nitrogen use efficiency. Contrarily, too much water and nitrogen input will make vegetative growth more vigorous than reproductive growth, delaying the ripening process of the fruit and reducing yield and water and nitrogen use efficiency (Shah et al., 2017b). The results of this study demonstrated that water and nitrogen input have an important impact on apple yield, IWUE, TWUE, and NPFP. The yield initially increased and then decreased with the increase of irrigation amount and nitrogen application rate (Figure 4), IWUE diminished with the increase of irrigation amount (Figures 5A,B), and NPFP diminished with the boost of nitrogen application rate (Figures 5E,F). The amount of nitrogen applied has no major effect on IWUE and TWUE under FI and DI_L_. Water and fertilizer coupling has a threshold response. When the input of water and fertilizer is less than the threshold, it will result in a crop yield reduction. Conversely, when the input of water and nitrogen is greater than the threshold, an increase in crop yield is not apparent and it may even dwindle (Zhou et al., 2015; Wang Z. H. et al., 2018). In this study, the DI_L_N_M_ treatment had the greatest apple yield, while the DI_S_N_L_ treatment had the least yield (Figure 4). This could be due to an extreme water deficit reducing the chemical activity of the water, ensuing in a major drop in cell turgor, leaf stomata closure, photosynthesis weakness, cell growth hindrance, root growth inhibition, an increase in xylem sap flow viscosity, and reduction in the improvement of the absorption and transportation capacity of soil nutrients (Hao et al., 2017). Low-nitrogen application is unable to make up for the lack of nutrients in the tree, resulting in the lack of improvement in water and nitrogen productivity.

### Coupling Effects of Water and Nitrogen on Apple Quality

The fruit’s commodity value is directly affected by its quality and is the main measurement index of competition in the fruit market. Water is the media and medium through which fruit quality is improved. Proper water stress during different growth periods of crops is able to control the plant metabolism, advance the absorption, transportation, and transformation of inorganic and organic substances, promote the accumulation of photosynthetic products, and improve fruit quality (Zhou et al., 2015; Hao et al., 2019). Nitrogen content is the main factor in the formation of fruit quality. Proper nitrogen application can improve the content of soluble sugar and vitamin C in fruit (Wang et al., 2016; Ren et al., 2019), increase the fruit shape index and peel brightness (Zhang et al., 2021), and reduce the content of titratable acid (Yang et al., 2018). In this study, it was found that the soluble sugar content increased with an increase in the irrigation quantity, while the opposite development was discovered for the titratable acid content (Table 2). The dissimilar water supply situation may have changed the plant source–sink relationship (Rodrigues et al., 2019), altering the degree of hydrolysis of the protein, starch, fat, and other components of the fruit. An extreme water deficit reduced the vitamin C content significantly (Table 2), which was likely due to excessive water stress reducing the physiological activity of the fruit trees, resulting in a lower activity of key enzymes for vitamin C synthesis (Guo et al., 2011).

Fruit firmness increased with increasing severity of the water deficit (Table 2) due to drought stress changing the physiological mechanism of fruit softening, restricting the expansion and division of pulp cells, and increasing the density of pulp cells (Hao et al., 2017; Romero and Rose, 2019; Zhong et al., 2019). The sugar–acid ratio and color index under FI had no major difference compared with DI_L_, but was appreciably higher than DI_M_ and DI_S_ (Table 2), which is a different result from that of Gelly et al. (2004), who found that deficit irrigation considerably increased the sugar–acid ratio in peaches and resulted in the fruit being a ruddier color. The explanation for the findings of this study may be that apples (*Hanfu*) mature comparatively later in Northern Shaanxi, and some rainy weather occurred at some point in the fruit expansion stage (stage III) of Hanfu in 2019 and 2020, meaning the fruit received inadequate temperature and sunshine hours during the fruit maturity stage (stage IV). The end of stage IV is a vital period for fruit sugar-acid conversion and coloring, so in the end, deficit irrigation failed to appreciably improve the fruit sugar-acid ratio and coloring index.

### Multi-Objective Decision Making and Evaluation on Improved TOPSIS Method

The TOPSIS method has been extensively used in the development of crop irrigation and fertilization methods, by assessing the relative pros and cons according to the proximity of limited evaluation objects to idealized objectives. Wang H. D. et al. (2019) used the TOPSIS method to appraise the water and fertilizer production efficiency of potatoes. The limited productivity of fertilizer was affected by the fertilizer quantity, which made the distribution data biased, and it was hard to balance with other evaluation indexes. Therefore, the partial productivity of fertilizer was not taken into account in the comprehensive evaluation. The weighted TOPSIS method was utilized to suggest the water and fertilizer strategy for high yield and quality of pepper by Xiang (2017). The yield, quality, water use efficiency, and fertilizer partial productivity were used as the evaluation indexes, and the weights were 0.25. The weights of the four quality indexes were also the same (0.25/4 = 0.0625). Although Xiang (2017) set the weight to each evaluation index, these are all based on biased judgment. Additionally, the partial productivity of fertilizer in this paper is biased distribution. The direct analysis will lead to the evaluation results approaching to the processing with the largest value of the index, which will inevitably affect the scientificity and rationality of comprehensive appraisal. Liu et al. (2021) used the weighted TOPSIS technique to verify the optimal irrigation and fertilization mode based on mango yield, quality, and IWUE and utilized the entropy weight method to impartially weigh all the evaluation indexes. However, this will end up with a decrease in the weight of yield and IWUE with an increase in the number of quality indexes, i.e., the importance of yield and IWUE becomes weaker with an increase in the number of quality indexes.

In the wide-ranging evaluation based on crop yield, quality, water and fertilizer utilization, since the evaluation index is a skewed distribution or the weight between indicators is complicated to balance, the majority of scholars only based on a similar type of indicators (such as quality indicators) for comprehensive appraisal, but do not consider the biased distribution index in the evaluation process, even if the index is very important (Hao et al., 2019; Wang H. D. et al., 2019). In order to solve this predicament, in the comprehensive evaluation of this study, each evaluation index was tested for normality, and the index of bias distribution (IWUE and NPFP) was transformed into approximate normal distribution by the logarithmic transformation method. The game theory was used to integrate the subjective and objective weights by the analytic hierarchy process and coefficient of variation method. After that, the weighted TOPSIS method was used to comprehensively evaluate the yield, quality, and water and nitrogen use efficiency of apples. The study found that the highest comprehensive evaluation index was DI_L_N_M_ treatment, and the lowest was DI_S_N_L_ treatment (Table 5). Founded on the straightforward improvement of the TOPSIS method, this paper enlarges and improves the preceding works. The application of this method be is capable of making the comprehensive evaluation method even more scientific and germane.

### Regression Model of Comprehensive Evaluation Index With Water-Nitrogen Inputs

Through the numerical simulation of water and fertilizer input and crop yield, quality of water–fertilizer productivity, and the best water and fertilizer ratio of crops put forward by researchers (Patras et al., 2011; Xing et al., 2015), preceding studies have discovered that crop yield, quality, and water–fertilizer productivity cannot reach the maximum at the same time; and it is hard to be in the acceptable area of the same confidence interval (the interaction area is too small or there is no interaction area), so some appraisal indexes were discarded artificially in the comprehensive evaluation (Xing et al., 2015; Hao et al., 2019; Wang H. D. et al., 2019; Shi et al., 2022). In this study, the comprehensive evaluation index based on apple yield, quality, water use efficiency, and NPFP was acquired by improving the combined weight TOPSIS method of game theory (Table 5). Subsequently, the binary quadratic regression model of water and nitrogen input and comprehensive evaluation index was ascertained (Figure 6), and the value of comprehensive evaluation index in satisfactory areas of different confidence intervals was solved by MATLAB. The outcome showed that the irrigation and nitrogen applications corresponding to the maximum value of the comprehensive evaluation index were 106.18 mm and 522.27 kg ha^–1^ in 2019, and 106.00 mm and 536.12 kg ha^–1^ in 2020, respectively. The 2-year experiment demonstrated that the public intervals of irrigation interval and nitrogen interval where the comprehensive evaluation index achieved the utmost value of 99% were 95.76–116.59 mm and 471.56–575.75 kg ha^–1^, respectively (Figure 6). Thus, the recommended irrigation quantity for mountain apples in the Loess Plateau was 95–115 mm, and the recommended nitrogen amount was 470–575 kg ha^–1^ from the viewpoint of saving water and nitrogen, improving quality, and increasing yield.

## Conclusion

In the present study, the effects of irrigation level and nitrogen level on apple yield, IWUE, total TWUE, NPFP, and quality were significant. The apple yield and TWUE initially increased and after that decreased with increasing irrigation amount and nitrogen amount, the IWUE decreased with an increase in the irrigation amount, the NPFP decreased with an increase in the nitrogen amount. In this research, the game theory combined with the weights TOPSIS method was utilized to comprehensively appraise apple yield, quality, and water-nitrogen use efficiency. A binary quadratic regression equation was established between water-nitrogen inputs and comprehensive evaluation index, and the correct results demonstrated that the comprehensive evaluation index for apples showed an opening downward paraboloid with the input of irrigation-nitrogen amount. Taking into consideration the comprehensive benefit of water and nitrogen saving, elevated production, and superior quality, the recommended irrigation amount was 95–115 mm, and the recommended nitrogen amount was 470–575 kg ha^–1^. The outcomes of this study will possibly provide a theoretical basis for the scientific research of SRI and the supervision of mountain apple tree irrigation and fertilization in the Loess Plateau, China.

## Data Availability Statement

The original contributions presented in the study are included in the article/supplementary material, further inquiries can be directed to the corresponding author/s.

## Author Contributions

LF and XW conceived the study. KH, FJ, and LL collected the data and led the writing of the manuscript. KH, YP, XL, DW, SK, and XW participated in data interpretation and revised the manuscript. KH prepared the figures. All authors have read and approved the manuscript for publication.

## Conflict of Interest

The authors declare that the research was conducted in the absence of any commercial or financial relationships that could be construed as a potential conflict of interest.

## Publisher’s Note

All claims expressed in this article are solely those of the authors and do not necessarily represent those of their affiliated organizations, or those of the publisher, the editors and the reviewers. Any product that may be evaluated in this article, or claim that may be made by its manufacturer, is not guaranteed or endorsed by the publisher.
